# Aggregation-Induced Fluorescence of Carbazole and *o*-Carborane Based Organic Fluorophore

**DOI:** 10.3389/fchem.2019.00768

**Published:** 2019-11-15

**Authors:** Jiemin Jiao, Jia-Xin Kang, Yanna Ma, Qianyi Zhao, Huizhen Li, Jie Zhang, Xuenian Chen

**Affiliations:** ^1^School of Chemistry and Chemical Engineering, Henan Key Laboratory of Boron Chemistry and Advanced Energy Materials, Henan Normal University, Xinxiang, China; ^2^College of Chemistry and Molecular Engineering, Zhengzhou University, Zhengzhou, China

**Keywords:** carbazole, carborane, fluorescence, AIE, donor-acceptor system

## Abstract

Carbazole based fluorophores 9-butyl-3,6-bis-(phenylethynyl)-9H-carbazole (1) and 9-butyl-3,6-bis-(2-phenyl-*o*-carborane)-9H-carbazole (2) were synthesized *via* Sonogashira type cross-coupling reaction and followed by insertion with decaborane. Compound 1 exhibited far more intense fluorescence than 2 in THF solution, while in solid state 2 exerted stronger fluorescence than 1. The fluorescence quenching behavior of 2 in THF solution could be attributed to the intramolecular charge transfer of donor-acceptor system in 2, which was confirmed by electrochemical experiments and DFT calculations. The fluorescence enhancement of 2 in solid state can be ascribed to aggregational induced packing which was evidenced by crystallographic study. In addition, compound 2 showed typical aggregation induced emission (AIE) behavior.

## Introduction

Organic π-conjugated compounds have attracted much attention in the realm of functional material science for over a few decades because of their intrinsic flexibility on structural designing and functional modifying, as well as low cost and sustainable manufacturing potential in contrast to metal (especially rare-earth metal) based materials (Metzger, 1999; Beverina and Pagani, 2014). Among the organic functional π-conjugated materials, push-pull type chromophores with electron donating (donor) and accepting (acceptor) functional groups are of growing interests for their promising electro-optical properties and straightforward designing strategy (Kivala and Diederich, 2009). Introducing strong donor and acceptor into π-conjugated molecule structure results in complicated photo- and electronic- behaviors, leading to the discovery of various functional materials which could be applied in the field of organic light-emitting diodes (OLED) (Grimsdale et al., 2009; Xiao et al., 2011), semiconducting materials (Zhang et al., 2017), and organic solar cells (Gunes et al., 2007; Cheng et al., 2009; Mishra and Bauerle, 2012).

Carbazole demonstrates stability toward thermal, chemical, and environmental factors, and is also known for its excellent electron donating and charge transporting properties. Meanwhile, carbazole building block is easy to be functionalized at either nitrogen or aromatic backbones. With all these advantages, carbazole has been widely used and studied in photoluminescent and electronic materials (Grazulevicius et al., 2003; Kundu et al., 2003; Grigalevicius, 2006; Blouin and Leclerc, 2008; Karpicz et al., 2010; Chen et al., 2011; Li et al., 2012; Ooyama et al., 2012; Uoyama et al., 2012; Nishimoto et al., 2014; Yang et al., 2016; Wex and Kaafarani, 2017; Meier et al., 2019). *o*-Carborane (1,2-dicarba-*closo*-dodecaborane) hosts electronic deficient property due to the uniquely delocalized 3-center-2-electron bond on its electron withdrawing icosahedron boron cluster skeleton, and possesses highly polarized σ-aromatic character (Naito et al., 2015; Cho et al., 2016; Wu X. Y. et al., 2018). These features allow *o*-carborane cage to act as an acceptor through π-conjugated system, and the molecule exhibits interesting photoelectronic properties (Li et al., 2016; Nishino et al., 2016; Núñez et al., 2016; Naito et al., 2017). In the last few years, many works have been focused on donor-acceptor type π-conjugated compounds based on carbazole and *o*-carborane moieties due to the fore-mentioned advantages, and some of them are inspiring (Kwon et al., 2014; Furue et al., 2016). Recently, Yan's group reported a serial of carboranyl carbazoles exhibiting both aggregational induced emission (AIE) and electrochemical luminescence behavior in aqueous solution, which has strong potential for biochemical and diagnostic applications (Wei et al., 2019).

Researches on AIE have thrived since its discovery in 2001 (Luo et al., 2001), because this type of compounds exert excellent photoelectronic behaviors in condensed or solid state rather than solution, make it possible and convenient to design novel organic photoelectronic devices such as chemo- or bio-sensor, OLED, organic field-effect transistor (Hong et al., 2009, 2011; Ding et al., 2013; He et al., 2015). Herein, we report a carbazole and carborane based organic fluorophore which exhibits typical AIE behavior.

## Results and Discussion

### Synthesis

**Scheme 1:**
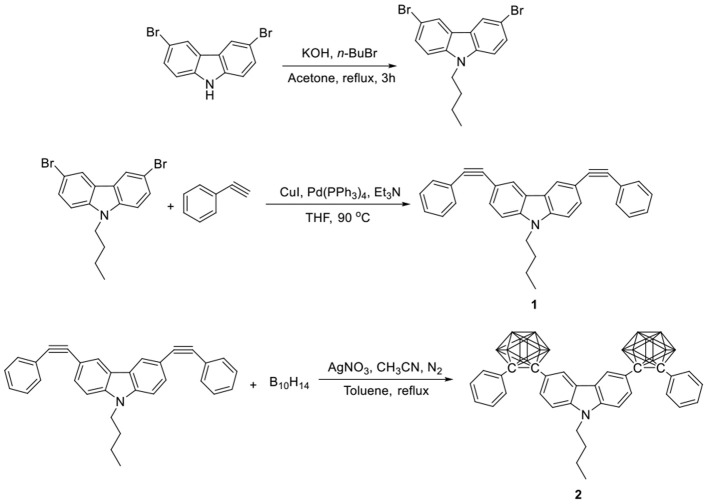
Synthetic procedures for **1** and **2**.

The synthetic procedures are shown in Scheme 1. According to the literature methods (Toppino et al., 2013; Zhu et al., 2016), 3,6-dibromo-9-butylcarbarzole was *N*-alkylated in the presence of potassium hydroxide, and then followed by Sonogashira type cross-coupling reaction with phenylacetylene to afford 9-butyl-3,6-bis-(phenylethynyl)-9H-carbazole (**1**). 9-butyl-3,6-bis-(2-phenyl-*o*-carborane)-9H-carbazole (**2**) was prepared by the insertion reaction of **1** and decaborane under a refluxing condition in toluene/CH_3_CN in the presence of a catalytic amount of silver nitrate. As a new compound, **2** was characterized by NMR, IR, and X-ray crystallography (see Figures S1–S7, Table S1, and Check CIF Report in Supplementary Material).

### FT-IR and UV-vis Spectra

**Figure 1:**
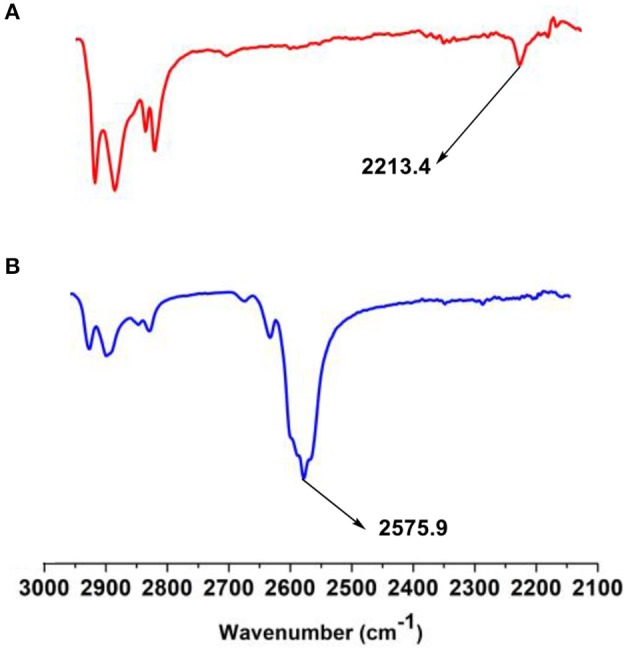
FT-IR spectra of **(A)** compound **1** and **(B)** compound **2** in 2,100–3,000 cm^−1^.

In the FT-IR spectrum of **1** (Figure 1), the absorption at 2,213 cm^−1^ is considered to be the C-C triple bond absorption, which disappeared in the FT-IR spectrum of **2**, indicating the insertion reaction of decaborane and alkyne moiety of **1**. Also, the appearance of strong absorption of B-H vibration at 2,576 cm^−1^ confirmed the existence of B-H bond in compound **2**, indicating the conversion from **1** to **2** (Yin et al., 2018).

**Figure 2:**
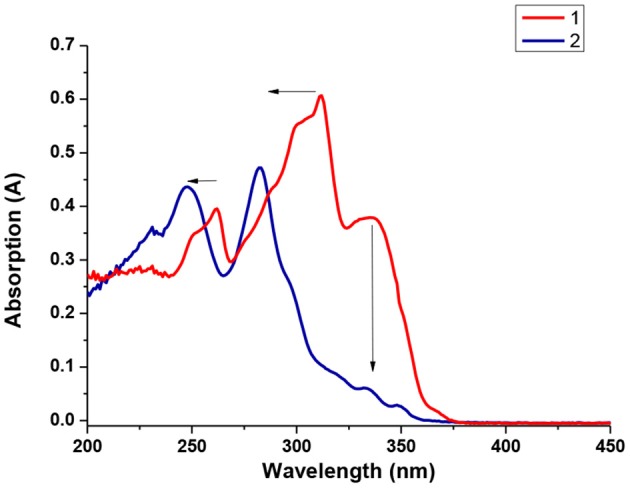
UV-vis spectra of **1** and **2** in THF solution at 1.0 × 10^−5^ mol/L.

The UV-vis absorption spectra of **1** and **2** were recorded in THF solution. As shown in Figure 2, the absorption band at 260, 310, and 330 nm could be assigned to the n-π and n-π^*^ transition of electron-enriched carbazole (Cz) moiety to the phenylacetylene fragment of **1** (Li et al., 2012; Gong et al., 2014), while blue shift was observed for the absorption band at 260 and 310 nm of compound **2**. This blue shift effect could be attributed to the wider HOMO/LUMO energy gap on Cz fragment caused by the highly electron-deficient *o*-carborane (Cb) moiety of **2** (Yokoyama et al., 2018). Moreover, the formation of Cb cluster in **2** leads to the disruption of donor-linker-acceptor conjugated system (Cz-phenylacetylene), hence the absorption band of intramolecular charge transfer transition at 330 nm is highly reduced (Wang et al., 2018; Wu Q. J. et al., 2018), as evidenced in Figure 2.

### Fluorescence Behaviors

**Figure 3:**
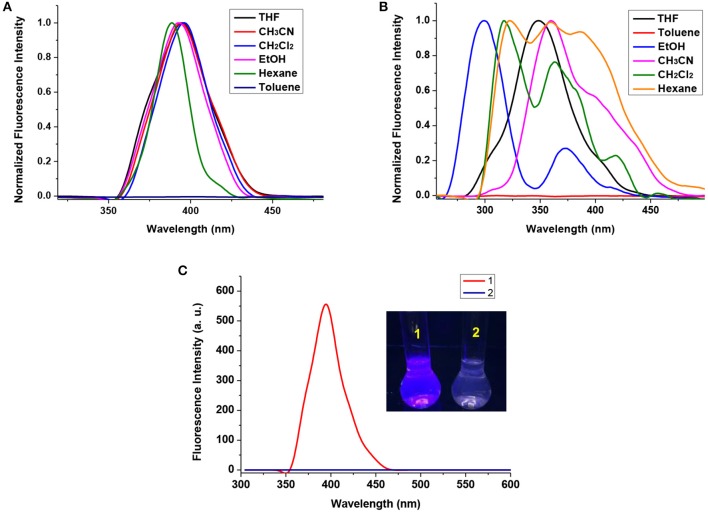
**(A)** Normalized fluorescence spectra of **1** in different solvents; **(B)** Normalized fluorescence spectra of **2** in different solvents; **(C)** Fluorescence spectra of **1** and **2** in THF solution at 1.0 × 10^−5^ mol/L. Inset: photograph of **1** and **2** under a 365 nm UV lamp.

The fluorescence spectra of compound **1** and **2** in various of solutions were recorded, and THF was chosen as solvent for the investigation of the solution excited behaviors. Upon excitation at 304 nm, compound **1** exhibits intense fluorescent emission at around 395 nm in THF solution, and the stokes shifts, which are determined by the difference in the wavelength between position of the band maxima of the excitation and emission spectra of the same electronic transition were almost identical in different solvents (Figure 3A and Figure S8). In the case of compound **2**, significant fluorescence quenching effect was observed (Figure 3C). Also, in various solutions of **2** at higher concentration, the fluorescence emissions vary from 299 to 360 nm upon excitation at 255 nm (Figure 3B and Figure S9), indicating the different photoluminescence mechanism. The intense blue fluorescent emission of compound **1** can be assigned to the local excited-state emission of a Cz-phenylacetylene fragment (Li et al., 2019), and the solvatochromic shift of **2** suggested the intramolecular charge-transfer (ICT) behavior between Cb and Cz units (Wee et al., 2012a; Nghia et al., 2018b). The temperature dependency of emission of **2** was also investigated (Figure S10), the results showed that the fluorescence intensity of **2** slightly decreased when temperature rises, implying that the emission band at around 348 nm was from twisted-ICT state, whose emission effect could be affected by environment conditions such as polarity of solvent and temperature (Nishino et al., 2017).

**Figure 4:**
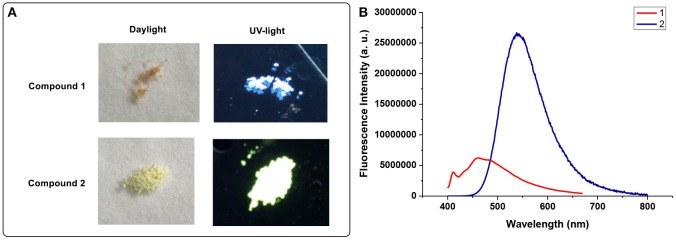
**(A)** Photograph of compound 1 and 2 under daylight and 365 nm UV lamp, respectively. **(B)** Fluorescence spectra of 1 and 2 in solid powder state.

Solid state fluorescence behaviors of compound **1** and **2** are also studied, detailed data are listed in Table 1. As shown in Figure 4, compound **1** exerts fluorescence at 455 nm with a shoulder peak situated at 410 nm, and compound **2** shows a relatively sharp single peak at 550 nm. For compound **1**, the major emission band at 455 nm could be assigned to the n-π and π-π^*^ local excited state emission of Cz-phenylacetylene fragment, and the minor shoulder emission peak at 410 nm comes from the isolated emission of Cz moiety (Li et al., 2019). Time-resolved measurement (Figures S11, S12; Tables S2, S3) also confirmed the bi-exponential decay behavior of **1**, indicating the double excited state. It is interesting that **2** exhibits stronger fluorescent emission than **1** in solid state, whereas in THF solution the fluorescent emission of **2** is nearly quenched completely. This phenomenon could be related to the photoluminescence mechanism for compound **1** and **2**. As we formerly discussed, in THF solution the fluorescent emission of **1** comes from the local excited-state emission of a Cz-phenylacetylene conjugated fragment, which no longer exists in **2** because of the introduction of Cb moiety (Nghia et al., 2018a). Also, the ICT quenching effect caused by the highly electron-deficient Cb inhibits the fluorescent emission of **2** (Wu Q. J. et al., 2018). Hence in THF solution, compound **1** exerts a much stronger fluorescence intensity than **2**.

**Table 1:** Photo-physical datas of **1** and **2**.

**Compound**	**λ_abs_/nm^[a]^**	**λ_ex_/nm**		**λ_em_/nm**		**τ_1_ /ns^[b]^**	**τ_2_ /ns^[b]^**	**PLQY**	
		**[THF]**	**[solid]**	**[THF]**	**[solid]**			${\Phi_{\text{F}}}^{\text{c}}$	${\Phi_{\text{F}}}^{\text{d}}$
**1**	260/310/330	304	399	395	462	1.52	4.18	0.34	0.15
**2**	247/280	255	399	348	538	5.59	−	0.03	0.56

^a^*Collected in N_2_-satuated THF solution at 1.0 × 10^-5^ mol/L under room temperature. ^b^Collected in solid state. Photoluminescence quantum yields taken in ^c^THF solution at 1.0 × 10^-5^ mol/L and ^d^solid state*.

### X-ray Crystallographic Studies

**Figure 5:**
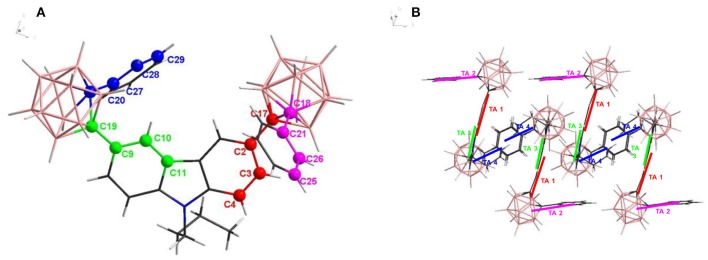
**(A)** X-ray crystallographic structure of compound **2**. Carbon atoms are colored in red, purple, green, and blue to put emphasis on torsion angles; **(B)** X-ray crystallographic packing structure of compound **2**. Short bars are painted in accordance with colored atoms, representing bonds of torsion angles to explain their spatial location in crystal structure.

The fluorescence enhancement of compound **2** in solid state is considered to be affected by the aggregational packing in its solid state. The X-ray crystallographic analysis of **2** indicated that two phenyl groups and one Cz unit adopted a Z-shaped arrangement. The C-C bond lengths of two Cb units are without much distinction (1.736 Å for C17-C18 and 1.733 Å for C19-C20, Figure 5A) due to the rigidity of Cb cluster. Nevertheless, the torsion angles around the carbon atoms of Cb explain their environmental difference in detail. The colored carbon atoms and C-C bonds in Figure 5A are from the Cb-phenyl units (including phenyl unit in Cz), they can also be considered as carbon atoms linked to phenyl units as well, and their torsion angles shall be 180° in a theoretically free environment without spatial hinderance. As shown in Table 2, the torsion angles of C17 and C18 are relatively smaller than C19 and C20, suggesting that Cb (C17-C18) is in a more distorted environment than Cb (C19-C20), which also indicates that there is a tightly packing style in solid state of compound **2** (Figure 5B). In THF solution of **2**, the rapid charge transfer from the fluorophore of Cz to the electron-withdrawing units of Cb weakens its fluorescent emission, while in solid state of **2**, intramolecular vibration and rotation are greatly restricted due to its tightly packing structure, which blocks the non-radiative pathway of excited state annihilation and opens up the radiative channel, thus its solid state fluorescent emission is enhanced (Zhu et al., 2008). Moreover, the prolonged fluorescence life of **2** also confirmed this restriction effect.

**Table 2:** Torsion angle in compound 2.

**Label**	**A-B-C-D**	**Torsion Angle (°)**
TA 1	C17-C2-C3-C4	171.3
TA 2	C18-C21-C26-C25	173.4
TA 3	C19-C9-C10-C11	177.9
TA 4	C20-C27-C28-C29	176.2

*TA, Torsion Angle*.

### AIE Studies

**Figure 6:**
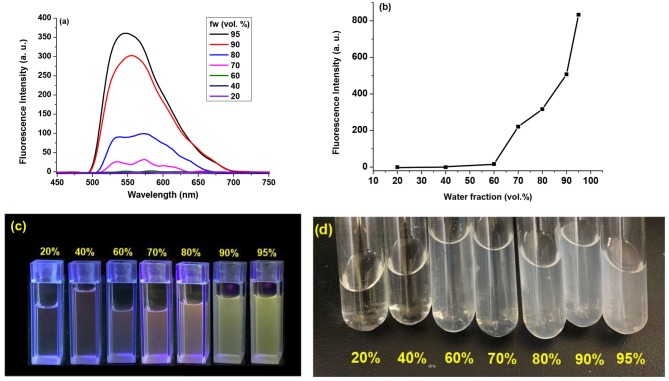
**(a)** Fluorescence spectra of compound **2** in THF/H_2_O solution (1.0 × 10^−5^ mol/L) with various H_2_O volume fractions. λ_ex_ = 212 nm. **(b)** Plot of emission intensity of compound **2** (at λ_em_ = 453 nm) in THF/H_2_O solution (1.0 × 10^−5^ mol/L) vs. increasing H_2_O volume fraction. **(c)** Photographs of compound **2** in THF/H_2_O solution (1.0 × 10^−5^ mol/L) with various H_2_O volume fractions, taken under 254 nm UV-light. **(d)** Photographs of compound **2** in THF/H_2_O solution (1.0 × 10^−5^ mol/L) with various H_2_O volume fractions, taken under daylight.

In order to further demonstrate the AIE behavior of compound **2**, the fluorescent emission spectra in THF/H_2_O solution with different volumetric ratios (fraction of water, or f_w_ in vol.%) were determined, and the results are shown in Figure 6. Compound **2** virtually showed weak fluorescent emission in either THF solution or THF/H_2_O solution with low H_2_O volume fraction. However, its fluorescent emission was significantly enhanced when f_w_ reaches 70%, and the emission intensity kept increasing as the f_w_ rises at higher levels. When f_w_ reached 95%, bright yellow fluorescence could be observed directly under commercially available UV lamp upon excitation at 254 nm. This typical AIE behavior could be ascribed to the decrease of solubility of **2** with the gradual addition of H_2_O. In THF solution, the fluorescence emission comes from the ICT emission and it is weak because the ICT quenching effect of the introduced carborane unit. As water fraction increased, the emission wavelength around 538 nm originating from the lowest energy ICT excited state was significantly enhanced with the formation of aggregation, which leads to the restriction of vibration and/or rotation of Cb and phenyl unit in compound 2. In the THF solution, the Cb and phenyl unit can rotate, resulting in the dissipation of the excitation energy as thermal energy. However, when the molecules aggregated, the rotation of the peripheral carborane and phenyl units are effectively impeded, which could not lead to non-radiative decay any longer, thus the molecules of 2 become strongly emissive (Furue et al., 2016). In fact, the THF/H_2_O solutions of **2** showed rather poor clarity at higher f_w_ level in contrast to its lower f_w_ solution, indicating that other than precipitation, this aggregation process was conducted at nanoscale (Liu et al., 2011).

### Electrochemical Behavior

**Figure 7:**
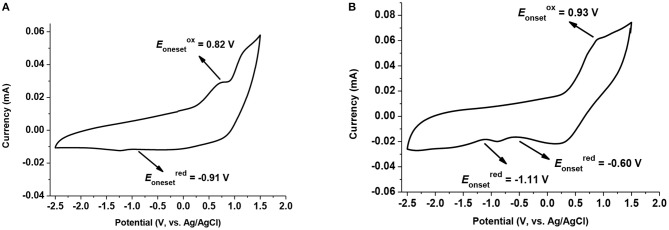
Cyclic voltammograms of **(A)** compound **1** and **(B)** compound **2** in dichloromethane using TBAF as conductive reagent. Scan rate: 100 mV/s. CV tests were carried out with a CHI760B electrochemical workstation in CH_2_Cl_2_ containing 0.1 M of sample and 0.1 M of Bu_4_NClO_4_ under an inert atmosphere with a glassy carbon working electrode, a platinum counter electrode, an Ag/AgCl reference electrode and ferrocene/ferrocenium as external reference.

Furthermore, cyclic voltammograms (CV) were also collected for both **1** and **2** by using dichloromethane with tetrabutylammonium perchlorate (TBAF), as reported in the literature for illustration on their electrochemical properties (Hosoi et al., 2011). The CV curve of **1** (Figure 7A) shows an oxidation peak at 0.82 V and a reduction peak at −0.91 V, might be ascribed to the electrochemical redox process on nitrogen atom in Cz skeleton (Kundu et al., 2003; Naito et al., 2018). In the case of **2** (Figure 7B), one more reduction peak of Cbs was recorded at −1.11 V, and redox peaks of Cz shifted to higher voltage (from 0.82/−0.91 to 0.93/−0.60), this is supposed to be the electron withdrawing effect of Cbs, making the oxidation process harder by dispersing the electron density on Cz skeleton, and confirming the donor-acceptor behavior in the structure of **2**.

### DFT Calculation

**Figure 8:**
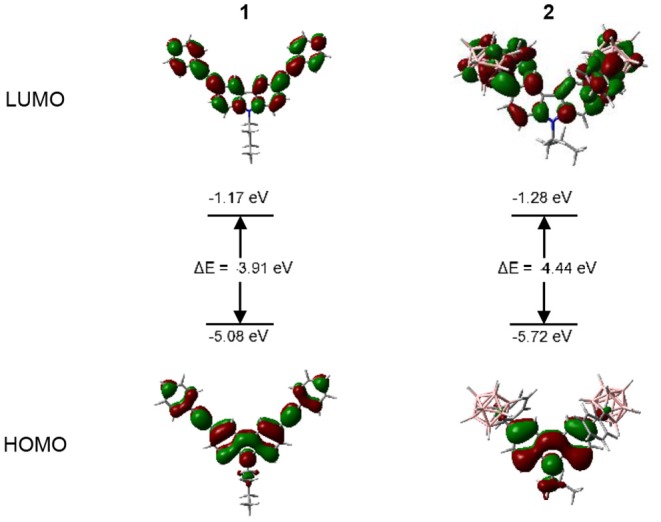
Frontier molecular orbitals and their energy levels of **1** and **2**, calculated by using B3LYP/6-311G(d,p) basis set.

DFT calculations were performed to determine the HOMO and LUMO energy levels of compound **1** and **2**. The Results show that the introduction of *o*-Cb units lowered both the HOMO and LUMO energy level (from −1.17 to −1.28 eV for LUMO, and from −5.08 to −5.72 eV for HOMO), this could be attributed to the electron-withdrawing ability of the *o*-Cb groups (Kokado and Chujo, 2009; Kokado et al., 2010; Wee et al., 2012b, 2013; Guo et al., 2015; Wu X. Y. et al., 2018). From Figure 8, it could be noted that the HOMO and LUMO were mainly located in the Cz-phenylethynyl conjugated skeleton for **1**, which is in accordance with the local excited state fluorescence emission. As for the case of 2, the LUMO distributed over the conjugated Cz-Cb skeleton, while its HOMO density was mainly delocalized in the Cz moiety, indicating that the conjugation was greatly reduced in contrast to **1** due to the donor-acceptor behavior between Cb and Cz, also caused the larger energy gap (from 3.91 to 4.44 eV) between the HOMO and LUMO level (Yin et al., 2018).

## Conclusion

In summary, two carbazole-based fluorophores have been synthesized, and their photo- and electrochemical behavior has been investigated. In THF solution, carbazole based fluorophore **1** exhibits intense blue fluorescence whereas the fluorescence of carbazole/carborane based fluorophore **2** is nearly quenched completely. However, **2** exerts much stronger fluorescence emission than **1** in solid state, and exhibits aggregational induced emission enhancement behavior. Crystallographic study confirmed the tightly packing style in the condensed state of **2**. Moreover, electrochemical experiments and DFT calculations were performed to explain the donor-acceptor behavior of the carbazole based fluorophores.

## Experimental Section

### General Experimental Conditions

All manipulations were performed under nitrogen atmosphere by using standard Schlenk techniques unless otherwise indicated. Chemical reagents except solvents were purchased via commercial sources and used without further purification. Tetrahydrofuran (THF) and toluene were refluxed on sodium-benzophenone system and freshly distilled prior to use. Triethylamine (Et_3_N) was refluxed on CaH_2_ and freshly distilled prior to use. ^1^H NMR spectra were recorded on Bruker AVANCE NEO 400 MHz, ^11^B, and ^13^C NMR spectra were recorded on Bruker AVANCE III HD 600 MHz, and CDCl_3_ were used as deuterated reagent unless otherwise specified. FT-IR spectra were recorded on BIO-RAD FTS-40. Solution fluorescence spectra were recorded on PerkinElmer Fluorescence Spectrometer LS55. Solid fluorescence spectra were recorded on Edinburgh Instrument FLS980. 3,6-dibromo-9-butylcarbarzole was synthesized according to reported literature (Zhu et al., 2016).

### Synthesis of 9-Butyl-3,6-bis(Phenylethynyl)-9H-Carbazole (1)

3,6-dibromo-9-butylcarbarzole (381.0 mg, 1.0 mmol), Pd(PPh_3_)_4_ (115.0 mg, 0.1 mmol), and CuI (20.0 mg, 0.1 mmol) were add to a Schlenk tube under inert atmosphere, and anhydrous THF (15 mL) and Et_3_N (15 mL) were inject into the system. The reaction mixture was stirred at ambient temperature for 10 min, then Phenylacetylene (245.0 mg, 2.4 mmol) was inject into the mixture and stirred overnight at 90°C. After the reaction was complete according to TLC monitoring, the reaction mixture was filtered through a thin layer of silica gel to afford lucid solution. After the solvents were removed under vacuum, the black viscous residue was diluted by CH_2_Cl_2_ and then washed with water and brine. The organic layers were collected and solvent was removed under reduced pressure, the crude product was subjected to chromatography column using n-hexane/ethyl acetate (v/v = 10/1) as elute to afford **1** as a yellow powder (173.4 mg, 41%). ^1^H NMR (400 MHz, *d*_6_-DMSO) δ(ppm): 8.50 (d, *J* = 1.0 Hz, 2H), 7.72–7.65 (m, 4H), 7.59–7.57 (dd, *J* = 8.4, 1.0 Hz, 4H), 7.47–7.41 (m, 6H), 4.45 (t, *J* = 7.1 Hz, 2H), 1.77 (p, *J* = 7.2 Hz, 2H), 1.32 (h, *J* = 7.6 Hz, 2H), 0.90 (t, *J* = 7.3 Hz, 3H).

### Synthesis of 9-Butyl-3,6-Bis(2-Phenyl-*o*-Carborane)-9H-Carbazole (2)

Decaborane (104.0 mg, 0.85 mmol) was dissolved in CH_3_CN (1.0 mL) under inert atmosphere, and then was stirred for 1 h at 60°C. After cooling to room temperature, **1** (105.0 mg, 0.25 mmol), AgNO_3_ (7.0 mg, 0.04 mmol) and dry toluene (20.0 mL) were add into the reaction system, and then refluxed for 2 days. After cooling, solvents were removed under vacuum, and the black residue was subject to silica gel chromatography using n-hexane as eluent to afford **2** as a yellow powder (37.9 mg, 23%) (Toppino et al., 2013). A yellow single crystal can be obtained by recrystallization from CHCl_3_ and n-hexane as combined solvents. ^1^H NMR (400 MHz, CDCl_3_) δ(ppm): 8.05 (d, J = 1.8 Hz, 2H), 7.49 (dd, J = 8.9, 2.2 Hz, 6H), 7.18–7.04 (m, 8H), 4.05 (t, J = 7.2 Hz, 2H), 3.40–1.83 (w, 20H), 1.66 (p, J = 7.3 Hz, 2H), 1.25–1.19 (m, 2H), 0.87 (t, J = 7.3 Hz, 3H). ^13^C (600 MHz, CDCl_3_) δ(ppm): 159.3, 140.3, 132.9, 129.6, 124.0, 122.5, 116.0, 114.1, 114.0, 109.0, 89.2, 87.7, 55.3, 43.1, 31.1, 20.5, 13.9. ^11^B NMR (600 MHz, CDCl_3_) δ(ppm): −3.1 (4B), −11.2 (6B). IR (KBr): (ν cm^−1^) 2,576 (B-H).

### Cyclic Voltammograms Condition

CV tests were carried out with a CHI760B electrochemical workstation in CH_2_Cl_2_ containing 0.1 M of sample and 0.1 M of Bu_4_NClO_4_ at scan rate of 100 mV/s^−1^ under a nitrogen atmosphere. A three-electrodes cell system was used, containing a glassy carbon working electrode, a platinum counter electrode and an Ag/AgCl reference electrode, respectively.

### Density Functional Calculations

The frontier molecular orbitals of the compounds were performed with the Gaussian 09W. Structural optimization was performed before the calculation of the HOMO/LUMO energies. The Becke3LYP(B3LYP) functional with the 6-311G(d, p) basis set was used for all calculations (Frisch et al., 2009).

### X-ray Structure Determination

X-ray diffraction data was collected on a Burker Smart CCD Apex DUO diffractometer with a graphite mono-chromated CuKα radiation (λ = 1.54184 Å) using the ω-2θ scan mode. The data were corrected for Lorenz and polarization effects. The structure was solved by direct methods and refined on *F*^2^ by full-matrix least-squares methods using SHELXTL-2000. All calculations and molecular graphics were carried out on a computer using the SHELX-2000 program package and Diamond software.

## Data Availability Statement

All datasets generated for this study are included in the article/Supplementary Material.

## Author Contributions

JJ performed the design, synthesis experiments, and collection of the data. J-XK and HL made contributions on DFT calculation. YM, QZ, and JZ made contribution on single crystal data interpretation. XC made substantial contribution on data analysis and manuscript writing.

### Conflict of Interest

The authors declare that the research was conducted in the absence of any commercial or financial relationships that could be construed as a potential conflict of interest.

## References

[B1] Beverina L. Pagani G. A. (2014). π-Conjugated zwitterions as paradigm of donor-acceptor building blocks in organic-based materials. Acc. Chem. Res. 47, 319–329. 10.1021/ar4000967 24087897

[B2] Blouin N. Leclerc M. (2008). Poly(2,7-carbazole)s: structure-property relationships. Acc. Chem. Res. 41, 1110–1119. 10.1021/ar800057k 18656967

[B3] Chen Z. Ono R. J. Wiggins K. M. Cui H. L. Rong C. R. Bielawski C. W. (2011). Synthesis and characterization of polyketones containing pendant carbazoles. Polymer 52, 1731–1737. 10.1016/j.polymer.2011.02.030

[B4] Cheng Y. J. Yang S. H. Hsu C. S. (2009). Synthesis of conjugated polymers for organic solar cell applications. Chem. Rev. 209, 5868–5923. 10.1021/cr900182s 19785455

[B5] Cho Y. J. Kim S. Y. Cho M. J. Han W. S. Son H. J. Cho D. W.. (2016). Aggregation-induced emission of diarylamino-pi-carborane triads: effects of charge transfer and pi-conjugation. Phys. Chem. Chem. Phys. 18, 9702–9708. 10.1039/C5CP07883K 26996491

[B6] Ding D. Li K. Liu B. Tang B. Z. (2013). Bioprobes based on AIE fluorogens. Acc. Chem. Res. 46, 2441–2453. 10.1021/ar3003464 23742638

[B7] Frisch M. J. Trucks G. W. Schlegel H. B. Scuseria G. E. Robb M. A. Cheeseman J. R. (2009). Gaussian 09W (Revision A.01). Wallingford CT: Gaussian Inc.

[B8] Furue R. Nishimoto T. Park I. S. Lee J. Y. Yasuda T. (2016). Aggregation-induced delayed fluorescence based on donor/acceptor-tethered janus carborane triads: unique photophysical properties of nondoped OLEDs. Angew. Chem. Int. Ed Engl. 55, 7171–7175. 10.1002/anie.201603232 27145481

[B9] Gong W. L. Wang B. Aldred M. P. Li C. Zhang G. F. Chen T. (2014). Tetraphenylethene-decorated carbazoles: synthesis, aggregation-induced emission, photo-oxidation and electroluminescence. J. Mater. Chem. C 2, 7001–7012. 10.1039/C4TC01019A

[B10] Grazulevicius J. V. Storhriegl P. Pielichowski J. Pielichowski K. (2003). Carbazole-containing polymers: synthesis, properties and applications. Prog. Polym. Sci. 28, 1297–1353. 10.1016/S0079-6700(03)00036-4

[B11] Grigalevicius S. (2006). 3,6(2,7),9-Substituted carbazoles as electroactive amorphous materials for optoelectronics. Synth. Met. 156, 1–12. 10.1016/j.synthmet.2005.10.004

[B12] Grimsdale A. C. Chan K. L. Martin R. E. Jokisz P. G. Holmes A. B. (2009). Synthesis of light-emitting conjugated polymers for applications in electroluminescent devices. Chem. Rev. 109, 897–1091. 10.1021/cr000013v 19228015

[B13] Gunes S. Neugebauer H. Sariciftci N. S. (2007). Conjugated polymer-based organic solar cells. Chem. Rev. 107, 1324–1338. 10.1021/cr050149z 17428026

[B14] Guo J. X. Liu D. Q. Zhang J. H. Zhang J. J. Miao Q. Xie Z. W. (2015). o-Carborane functionalized pentacenes: synthesis, molecular packing and ambipolar organic thin-film transistors. Chem. Commun. 51, 12004–12007. 10.1039/C5CC03608A 26121634

[B15] He Z. K. Shan L. Mei J. Wang H. Lam J. W. Y. Sung H. H. Y.. (2015). Aggregation-induced emission and aggregation-promoted photochromism of bis(diphenylmethylene)dihydroacenes. Chem. Sci. 6, 3538–3543. 10.1039/C5SC00900F 28717460 PMC5500900

[B16] Hong Y. N. Lam J. W. Y. Tang B. Z. (2009). Aggregation-induced emission: phenomenon, mechanism and applications. Chem. Commun. 4332–4353. 10.1039/b904665h 19597589

[B17] Hong Y. N. Lam J. W. Y. Tang B. Z. (2011). Aggregation-induced emission. Chem. Soc. Rev. 40, 5361–5388. 10.1039/c1cs15113d 21799992

[B18] Hosoi K. Inagi S. Kubo T. Fuchigami T. (2011). o-Carborane as an electron-transfer mediator in electrocatalytic reduction. Chem. Comm. 47, 8632–8634. 10.1039/c1cc12912k 21720623

[B19] Karpicz R. Kirkus M. Grazulevicius J. V. Gulbinas V. (2010). Fluorescence quenching by charge carriers in indolo[3,2-b]carbazole-based polymer. J. Lumin. 130, 722–727. 10.1016/j.jlumin.2009.11.029

[B20] Kivala M. Diederich F. (2009). Acetylene-derived strong organic acceptors for planar and nonplanar push-pull chromophores. Acc. Chem. Res. 42, 235–248. 10.1021/ar8001238 19061332

[B21] Kokado K. Chujo Y. (2009). Emission via aggregation of alternating polymers with o-carborane and p-phenylene-ethynylene sequences. Macromolecules 42, 1418–1420. 10.1021/ma8027358

[B22] Kokado K. Nagai A. Chujo Y. (2010). Poly(γ-glutamic acid) hydrogels with water-sensitive luminescence derived from aggregation-induced emission of o-carborane. Macromolecules 43, 6463–6468. 10.1021/ma100792z

[B23] Kundu P. Tomas K. R. J. Lin J. T. Tao Y. T. Chien C. H. (2003). High-Tg carbazole derivatives as blue-emitting hole-transporting materials for electroluminescent devices. Adv. Funct. Mater. 13, 445–452. 10.1002/adfm.200304308

[B24] Kwon S. N. Wee K. R. Cho Y. J. Kang S. O. (2014). Carborane dyads for photoinduced electron transfer: photophysical studies on carbazole and phenyl-o-carborane molecular assemblies. Chemistry 20, 5953–5960. 10.1002/chem.201304474 24805274

[B25] Li M. J. Peng F. Ying L. Xu J. K. (2019). Sky-blue fluorescent small-molecules with high quantum efficiency: synthesis, structures, AIE properties, and applications in solution-processed non-doped OLEDs. J. Mater. Chem. C 7, 3553–3559. 10.1039/C9TC00546C

[B26] Li X. Yan H. Zhao Q. (2016). Carboranes as a tool to tune phosphorescence. Chem. Eur. J. 22, 1888–1898. 10.1002/chem.201503456 26603358

[B27] Li Z. Lu J. Li S. D. Qin S. H. Qin Y. M. (2012). Orderly ultrathin films based on perylene/poly(N-vinyl carbazole) assembled with layered double hydroxide nanosheets: 2D fluorescence resonance energy transfer and reversible fluorescence response for volatile organic compounds. Adv. Mater. 24, 6053–6057. 10.1002/adma.201203040 22936625

[B28] Liu Y. Chen S. M. Lam J. W. Y. Lu P. Kwok R. T. K. Mahtab F. (2011). Tuning the electronic nature of aggregation-induced emission luminogens with enhanced hole-transporting property. Chem. Mater. 23, 2536–2544. 10.1021/cm2003269

[B29] Luo J. D. Xie Z. Lam J. W. Cheng H. Qiu C. Kwok H. S. (2001). Aggregation-induced emission of 1-methyl-1,2,3,4,5-pentaphenylsilole. Chem. Commun. 2001, 1740–1741. 10.1039/b105159h 12240292

[B30] Meier M. Ji L. Nitsch J. Krummenacher I. Deißenberger A. Auerhammer D.. (2019). Preparation and characterization of a pi-conjugated donor-acceptor system containing the strongly electron-accepting tetraphenylborolyl unit. Chemistry 25, 4707–4712. 10.1002/chem.201805454 30786077

[B31] Metzger R. M. (1999). Electrical rectification by a molecule: the advent of unimolecular electronic devices. Acc. Chem. Res. 32, 950–957. 10.1021/ar9900663

[B32] Mishra A. Bauerle P. (2012). Small molecule organic semiconductors on the move: promises for future solar energy technology. Angew. Chem. Int. Ed Engl. 51, 2020–2067. 10.1002/anie.201102326 22344682

[B33] Naito H. Morisaki Y. Chujo Y. (2015). o-Carborane-based anthracene: a variety of emission behaviors. Angew. Chem. Int. Ed Engl. 54, 5084–5087. 10.1002/anie.201500129 25729004

[B34] Naito H. Nishino K. Morisaki Y. Tanaka K. Chujo Y. (2017). Highly-efficient solid-state emissions of anthracene–o-carborane dyads with various substituents and their thermochromic luminescence properties. J. Mater. Chem. C 5, 10047–10054. 10.1039/C7TC02682J

[B35] Naito H. Uemura K. Morisaki Y. Tanaka K. Chujo Y. (2018). Enhancement of luminescence efficiencies by thermal rearrangement from ortho- to meta-carborane in bis-carborane-substituted acenes. Eur. J. Org. Chem. 2018, 1885–1890. 10.1002/ejoc.201800151

[B36] Nghia N. V. Jana S. Sujith S. Ryu J. Y. Lee J. Lee S. U.. (2018b). Nido-carboranes: donors for thermally activated delayed fluorescence. Angew. Chem. Int. Ed Engl. 57, 12483–12488. 10.1002/anie.201806922 30091167

[B37] Nghia N. V. Oh J. H. Sujith S. Jung J. H. Lee M. H. (2018a). Tuning the photophysical properties of carboranyl luminophores by closo- to nido-carborane conversion and application to OFF–ON fluoride sensing. Dalton Transac. 47, 17441–17449. 10.1039/C8DT03771J 30488927

[B38] Nishimoto T. Yasuda T. Lee S. Y. Kondoa R. Adachi C. (2014). A six-carbazole-decorated cyclophosphazene as a host with high triplet energy to realize efficient delayed-fluorescence OLEDs. Mater. Horiz. 1, 264–269. 10.1039/C3MH00079F

[B39] Nishino K. Hashimoto K. Tanaka K. Morisaki Y. Chujo Y. (2016). Synthesis and properties of highly-rigid conjugation system based on bi(benzo[b]thiophene)-fused o -carborane. Tetrahedron Lett. 57, 2025–2028. 10.1016/j.tetlet.2016.03.069

[B40] Nishino K. Uemura K. Gon M. Tanaka K. Chujo Y. (2017). Enhancement of aggregation-induced emission by introducing multiple o-carborane substitutions into triphenylamine. Molecules 22:E2009. 10.3390/molecules22112009 29156590 PMC6150215

[B41] Núñez R. Tarres M. Ferrer-Ugalde A. Fabrizi de Biani F. Teixidor F. (2016). Electrochemistry and photoluminescence of icosahedral carboranes, boranes, metallacarboranes, and their derivatives. Chem. Rev. 116, 14307–14378. 10.1021/acs.chemrev.6b00198 27960264

[B42] Ooyama Y. Yamaguchi N. Inoue S. Nagano T. Miyazaki E. Fukuoka H. (2012). Mechanofluorochromism of carbazole-type D–π-A fluorescent dyes. Tetrahedron 68, 529–533. 10.1016/j.tet.2011.11.012

[B43] Toppino A. Genady A. R. El-Zaria M. E. Reeve J. Mostofian F. Kent J.. (2013). High yielding preparation of dicarba-closo-dodecaboranes using a silver(I) mediated dehydrogenative alkyne-insertion reaction. Inorg. Chem. 52, 8743–8749. 10.1021/ic400928v 23829543

[B44] Uoyama H. Goushi K. Shizu K. Nomura H. Adachi C. (2012). Highly efficient organic light-emitting diodes from delayed fluorescence. Nature 492, 234–238. 10.1038/nature11687 23235877

[B45] Wang Z. Y. Feng Y. Q. Wang N. N. Cheng Y. X. Quan Y. W. Ju H. X. (2018). Donor-acceptor conjugated polymer dots for tunable electrochemiluminescence activated by aggregation-induced emission-active moieties. J. Phys. Chem. Lett. 9, 5296–5302. 10.1021/acs.jpclett.8b02087 30157647

[B46] Wee K. R. Cho Y. J. Jeong S. Y. Kwon S. N. Lee J. D. Suh I. H.. (2012b). Carborane-based optoelectronically active organic molecules: wide band gap host materials for blue phosphorescence. J. Am. Chem. Soc. 134, 17982–17990. 10.1021/ja3066623 23057809

[B47] Wee K. R. Cho Y. J. Song J. K. Kang S. O. (2013). Multiple photoluminescence from 1,2-dinaphthyl-ortho-carborane. Angew. Chem. Int. Ed Engl. 52, 9682–9685. 10.1002/anie.201304321 23881691

[B48] Wee K. R. Han W. S. Cho D. W. Kwon S. N. Pac C. J. Kang S. O. (2012a). Carborane photochemistry triggered by aryl substitution: carborane-based dyads with phenyl carbazoles. Angew. Chem. Int. Ed Engl. 51, 2677–2680. 10.1002/anie.201109069 22298500

[B49] Wei X. Zhu M. J. Cheng Z. Lee M. J. Yan H. Lu C. S.. (2019). Aggregation-induced electrochemiluminescence of carboranyl carbazoles in aqueous media. Angew. Chem. Int. Ed Engl. 58, 3162–3166. 10.1002/anie.201900283 30698911

[B50] Wex B Kaafarani B. R. (2017). Perspective on carbazole-based organic compounds as emitters and hosts in TADF applications. J. Mater. Chem. C 5, 8622–8653. 10.1039/C7TC02156A

[B51] Wu Q. J. Wang M. H. Cao X. D. Zhang D. Sun N. Wan S. G. (2018). Carbazole/α-carboline hybrid bipolar compounds as electron acceptors in exciplex or non-exciplex mixed cohosts and exciplex-TADF emitters for high-efficiency OLEDs. J. Mater. Chem. C 6, 8784–8792. 10.1039/C8TC02353K

[B52] Wu X. Y. Guo J. X. Quan Y. J. Jia W. Jia D. Z. Chen Y. (2018). Cage carbon-substitute does matter for aggregation-induced emission features of o-carborane-functionalized anthracene triads. J. Mater. Chem. C 6, 4140–4149. 10.1039/C8TC00380G

[B53] Xiao L. X. Chen Z. J. Xu B. Luo J. X. Kong S. Gong Q. H.. (2011). Recent progresses on materials for electrophosphorescent organic light-emitting devices. Adv. Mater. 23, 926–952. 10.1002/adma.201003128 21031450

[B54] Yang L. L. Wang J. P. Yang L. Zhang C. Zhang R. L. Zhang Z. P. (2016). Fluorescent paper sensor fabricated by carbazole-based probes for dual visual detection of Cu^2+^ and gaseous H_2_S. RSC Adv. 6, 56384–56391. 10.1039/C6RA10293J

[B55] Yin Y. H. Li X. Yan S. B. Yan H. Lu C. S. (2018). Tetraphenylethylene-carborane-tetraphenylethylene triad: influence of steric bridge on aggregation-induced emission properties. Chem. Asian J. 13, 3155–3159. 10.1002/asia.201801172 30133156

[B56] Yokoyama M. Inada K. Tsuchiya Y. Nakanotani H. Adachi C. (2018). Trifluoromethane modification of thermally activated delayed fluorescence molecules for high-efficiency blue organic light-emitting diodes. Chem. Commun. 54, 8261–8264. 10.1039/C8CC03425G 29989113

[B57] Zhang J. Xu W. Sheng P. Zhao G. Y. Zhu D. B. (2017). Organic donor-acceptor complexes as novel organic semiconductors. Acc. Chem. Res. 50, 1654–1662. 10.1021/acs.accounts.7b00124 28608673

[B58] Zhu H. T. Shi B. B. Chen K. X. Wei P. F. Xia D. Y. Mondal J. H.. (2016). Cyclo[4]carbazole, an iodide anion macrocyclic receptor. Org. Lett. 18, 5054–5057. 10.1021/acs.orglett.6b02500 27653011

[B59] Zhu L. N. Yang C. L. Qin J. G. (2008). An aggregation-induced blue shift of emission and the self-assembly of nanoparticles from a novel amphiphilic oligofluorene. Chem. Commun. 6303–6305. 10.1039/b815431g 19048136

