# Supplementary material for: Aggregation-Induced Fluorescence of Carbazole and o-Carborane Based Organic Fluorophore
Source: Front Chem. 2019 Nov 15;7:768. doi: 10.3389/fchem.2019.00768 (PMC6873483; doi:10.3389/fchem.2019.00768)

checkCIF/PLATON report

You have not supplied any structure factors. As a result the full set of tests cannot be run.

THIS REPORT IS FOR GUIDANCE ONLY. IF USED AS PART OF A REVIEW PROCEDURE FOR PUBLICATION, IT SHOULD NOT REPLACE THE EXPERTISE OF AN EXPERIENCED CRYSTALLOGRAPHIC REFEREE.

No syntax errors found.   CIF dictionary   Interpreting this report

Datablock: hy

```
Bond precision:  C-C = 0.0073 A          Wavelength=1.54184

Cell:          a=10.4511(2)      b=13.2462(2)     c=16.0310(2)
               alpha=81.564(1)   beta=83.322(1)   gamma=83.508(1)
Temperature:   200 K
```

	Calculated	Reported
Volume	2169.95(6)	2169.95(6)
Space group	P -1	P -1
Hall group	-P 1	-P 1
Moiety formula	2(C32 H45 B20 N), 2(C H Cl3), C Cl3	C H Cl3, 0.5(C Cl3), C32 H45 B20 N
Sum formula	C67 H92 B40 Cl9 N2	C33.50 H46 B20 Cl4.50 N
Mr	1676.88	838.44
Dx,g cm-3	1.283	1.283
Z	1	2
Mu (mm-1)	2.961	2.961
F000	861.0	861.0
F000’	865.82	
h,k,lmax	12,16,19	12,16,19
Nref	8697	8311
Tmin,Tmax		0.259,1.000
Tmin’		

```
Correction method= # Reported T Limits: Tmin=0.259 Tmax=1.000
AbsCorr = MULTI-SCAN

Data completeness= 0.956          Theta(max)= 73.293

R(reflections)= 0.1071( 6767)     wR2(reflections)= 0.2627( 8311)

S = 1.082                   Npar= 551
```

The following ALERTS were generated. Each ALERT has the format
test-name_ALERT_alert-type_alert-level.
Click on the hyperlinks for more details of the test.

Alert level B

```
PLAT260_ALERT_2_B Large Average Ueq of Residue Including      Cl1A      0.188 Check
```

Alert level C

```
DIFMN02_ALERT_2_C  The minimum difference density is < -0.1*ZMAX*0.75
            _refine_diff_density_min given =     -1.498
            Test value =     -1.275
DIFMN03_ALERT_1_C  The minimum difference density is < -0.1*ZMAX*0.75
            The relevant atom site should be identified.
DIFMX02_ALERT_1_C  The maximum difference density is > 0.1*ZMAX*0.75
            The relevant atom site should be identified.
PLAT053_ALERT_1_C Minimum Crystal Dimension Missing (or Error) ...     Please Check
PLAT054_ALERT_1_C Medium  Crystal Dimension Missing (or Error) ...     Please Check
PLAT055_ALERT_1_C Maximum Crystal Dimension Missing (or Error) ...     Please Check
PLAT082_ALERT_2_C High R1 Value ................................       0.11 Report
PLAT084_ALERT_3_C High wR2 Value (i.e. > 0.25) ...................     0.26 Report
PLAT097_ALERT_2_C Large Reported Max.  (Positive) Residual Density      1.65 eA-3
PLAT098_ALERT_2_C Large Reported Min.  (Negative) Residual Density     -1.50 eA-3
PLAT213_ALERT_2_C Atom C7              has ADP max/min Ratio .....      3.4 oblate
PLAT260_ALERT_2_C Large Average Ueq of Residue Including       Cl1      0.109 Check
PLAT340_ALERT_3_C Low Bond Precision on  C-C Bonds ..............    0.00729 Ang.
```

Alert level G

```
PLAT003_ALERT_2_G Number of Uiso or Uij Restrained non-H Atoms ...          2 Report
PLAT012_ALERT_1_G No       _shelx_res_checksum Found in CIF ......     Please Check
PLAT042_ALERT_1_G Calc. and Reported MoietyFormula Strings  Differ     Please Check
PLAT045_ALERT_1_G Calculated and Reported Z Differ by a Factor ...       0.50 Check
PLAT083_ALERT_2_G SHELXL Second Parameter in WGHT  Unusually Large      14.07 Why ?
PLAT153_ALERT_1_G The s.u.'s on the Cell Axes   are Equal ..(Note)     0.0002 Ang.
PLAT154_ALERT_1_G The s.u.'s on the Cell Angles are Equal ..(Note)      0.001 Degree
PLAT186_ALERT_4_G The CIF-Embedded .res File Contains ISOR Records          2 Report
PLAT300_ALERT_4_G Atom Site Occupancy of Cl1A       Constrained at        0.5 Check
PLAT300_ALERT_4_G Atom Site Occupancy of Cl0A       Constrained at        0.5 Check
PLAT300_ALERT_4_G Atom Site Occupancy of Cl3        Constrained at        0.5 Check
PLAT300_ALERT_4_G Atom Site Occupancy of C0AA       Constrained at        0.5 Check
PLAT302_ALERT_4_G Anion/Solvent/Minor-Residue Disorder (Resd  3  )       100% Note
PLAT343_ALERT_2_G Unusual sp?      Angle Range in Main Residue for        C17 Check
PLAT343_ALERT_2_G Unusual sp?      Angle Range in Main Residue for        C18 Check
PLAT343_ALERT_2_G Unusual sp?      Angle Range in Main Residue for        C19 Check
PLAT343_ALERT_2_G Unusual sp?      Angle Range in Main Residue for        C20 Check
PLAT367_ALERT_2_G Long?  C(sp?)-C(sp?) Bond  C17      - C18      .       1.74 Ang.
PLAT367_ALERT_2_G Long?  C(sp?)-C(sp?) Bond  C18      - C21      .       1.51 Ang.
PLAT367_ALERT_2_G Long?  C(sp?)-C(sp?) Bond  C19      - C20      .       1.73 Ang.
PLAT432_ALERT_2_G Short Inter X...Y Contact  Cl1A     ..C0AA             1.58 Ang.
                                                 2-x,2-y,-z  =      2_775 Check
PLAT432_ALERT_2_G Short Inter X...Y Contact  Cl0A     ..C0AA             2.05 Ang.
                                                 2-x,2-y,-z  =      2_775 Check
PLAT432_ALERT_2_G Short Inter X...Y Contact  Cl3      ..C0AA             1.62 Ang.
                                                 2-x,2-y,-z  =      2_775 Check
PLAT432_ALERT_2_G Short Inter X...Y Contact  C0AA     ..C0AA             1.09 Ang.
                                                 2-x,2-y,-z  =      2_775 Check
PLAT720_ALERT_4_G Number of Unusual/Non-Standard Labels .........          23 Note
PLAT773_ALERT_2_G Check long C-C Bond in CIF: C17      --C18             1.74 Ang.
PLAT773_ALERT_2_G Check long C-C Bond in CIF: C19      --C20             1.73 Ang.
```

```
PLAT779_ALERT_4_G Suspect or Irrelevant (Bond) Angle in CIF .... #         12 Check
              C0AA -CL0A -C0AA    1.555   1.555   2.775             32.10 Deg.
PLAT779_ALERT_4_G Suspect or Irrelevant (Bond) Angle in CIF .... #        477 Check
              C0AA -CL1A -C0AA    2.775   1.555   1.555             37.90 Deg.
PLAT779_ALERT_4_G Suspect or Irrelevant (Bond) Angle in CIF .... #        483 Check
              C0AA -CL3  -C0AA    1.555   1.555   2.775             40.40 Deg.
PLAT779_ALERT_4_G Suspect or Irrelevant (Bond) Angle in CIF .... #        490 Check
              CL1A -C0AA -CL3     2.775   1.555   2.775             38.50 Deg.
PLAT779_ALERT_4_G Suspect or Irrelevant (Bond) Angle in CIF .... #        496 Check
              CL3  -C0AA -CL1A    1.555   1.555   1.555             36.70 Deg.
PLAT860_ALERT_3_G Number of Least-Squares Restraints ............         12 Note
```

```
  0 ALERT level A = Most likely a serious problem - resolve or explain
  1 ALERT level B = A potentially serious problem, consider carefully
 13 ALERT level C = Check. Ensure it is not caused by an omission or oversight
 33 ALERT level G = General information/check it is not something unexpected

 10 ALERT type 1 CIF construction/syntax error, inconsistent or missing data
 22 ALERT type 2 Indicator that the structure model may be wrong or deficient
  3 ALERT type 3 Indicator that the structure quality may be low
 12 ALERT type 4 Improvement, methodology, query or suggestion
  0 ALERT type 5 Informative message, check
```

It is advisable to attempt to resolve as many as possible of the alerts in all categories. Often the minor alerts point to easily fixed oversights, errors and omissions in your CIF or refinement strategy, so attention to these fine details can be worthwhile. In order to resolve some of the more serious problems it may be necessary to carry out additional measurements or structure refinements. However, the purpose of your study may justify the reported deviations and the more serious of these should normally be commented upon in the discussion or experimental section of a paper or in the "special_details" fields of the CIF. checkCIF was carefully designed to identify outliers and unusual parameters, but every test has its limitations and alerts that are not important in a particular case may appear. Conversely, the absence of alerts does not guarantee there are no aspects of the results needing attention. It is up to the individual to critically assess their own results and, if necessary, seek expert advice.

Publication of your CIF in IUCr journals

A basic structural check has been run on your CIF. These basic checks will be run on all CIFs submitted for publication in IUCr journals (*Acta Crystallographica*, *Journal of Applied Crystallography*, *Journal of Synchrotron Radiation*); however, if you intend to submit to *Acta Crystallographica Section C* or *E* or *IUCrData*, you should make sure that full publication checks are run on the final version of your CIF prior to submission.

Publication of your CIF in other journals

Please refer to the *Notes for Authors* of the relevant journal for any special instructions relating to CIF submission.

PLATON version of 06/01/2019; check.def file version of 19/12/2018

Datablock hy - ellipsoid plot

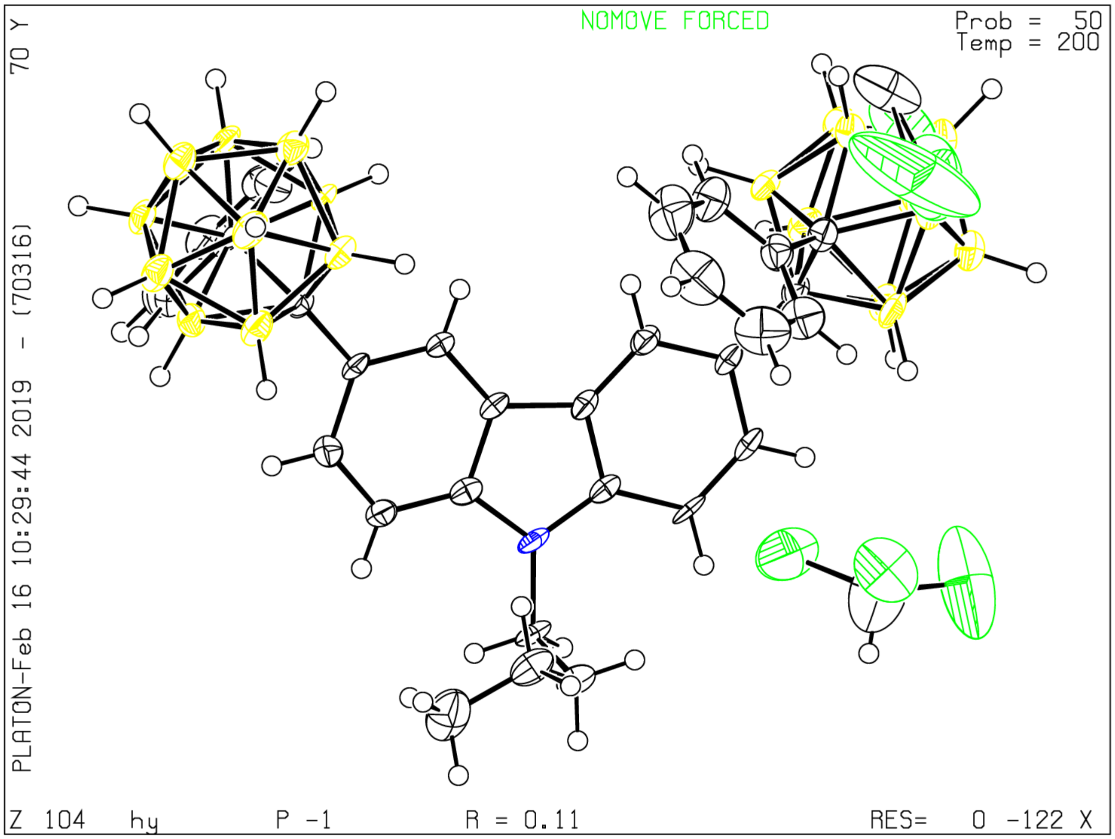

Supplement: Supplementary file 1 [file Data_Sheet_1.PDF]
