# Supplementary material for: Aggregation-Induced Fluorescence of Carbazole and o-Carborane Based Organic Fluorophore
Source: Front Chem. 2019 Nov 15;7:768. doi: 10.3389/fchem.2019.00768 (PMC6873483; doi:10.3389/fchem.2019.00768)
Supplement: Supplementary file 2 [file Data_Sheet_2.docx]

Supplementary Material

**1. General methods**

In this paper, all of the chemical reagents except solutions were purchased via commercial sources and used without further purification. Tetrahydrofuran (THF) and toluene were refluxed on sodium/benzophenone system and freshly distilled prior to use. Triethylamine (Et_3_N) was refluxed on CaH_2_ and freshly distilled prior to use. ^1^H NMR spectra were recorded on Bruke AVANCE NEO 400MHz, ^11^B and ^13^C NMR spectra were recorded on Bruke AVANCE Ⅲ HD600MHz, and CDCl_3_ were used as deuterated reagent unless specified. IR spectra were recorded on BIO-RAD FTS-40. Solution FL spectra were recorded on PerkinElmer Fluorescence Spectrometer LS55. Solid FL spectra were recorded on Edinburgh Instrument FLS980. 3,6-dibromo-9-butylcarbarzole was synthesized according to reported literature. [^1^](#_ENREF_1)

**2. Synthesis**

**1**. 3,6-dibromo-9-butylcarbarzole (381.0 mg, 1.0 mmol), Pd(PPh_3_)_4_ (115.0 mg, 0.1 mmol) and CuI (20.0 mg, 0.1 mmol) were add to a Schlenk tube under inert atmosphere, and anhydrous THF (15 mL) and Et_3_N (15 mL) were inject into the system. The reaction mixture was stirred at ambient temperature for 10 minutes, then Phenylacetylene (245.0 mg, 2.4 mmol) was inject into the mixture and stirred overnight at 90 ^o^C. After the reaction was complete according to TLC monitoring, the reaction mixture was filtered through a thin layer of silica gel to afford lucid solution. After the solvents were removed under vacuum, the black viscous residue was diluted by CH_2_Cl_2_ and then washed with water and brine. The organic layers were collected and solvent was removed under reduced pressure, the crude product was subjected to chromatography column using n-hexane/ethyl acetate (v/v=10/1) as elute to afford **1** as a yellow powder (173.4 mg, 41%). ^1^H NMR (400 MHz, *d*_6_-DMSO) δ(ppm): 8.50 (d, *J* = 1.0 Hz, 2H), 7.72-7.65 (m, 4H), 7.59-7.57 (dd, *J* = 8.4, 1.0 Hz, 4H), 7.47-7.41 (m, 6H), 4.45 (t, *J* = 7.1 Hz, 2H), 1.77 (p, *J* = 7.2 Hz, 2H), 1.32 (h, *J* = 7.6 Hz, 2H), 0.90 (t, *J* = 7.3 Hz, 3H).

**2**. Decaborane (104.0 mg, 0.85 mmol) was dissolved in CH_3_CN (1.0 mL) under inert atmosphere, and then was stirred for 1 h at 60 ^o^C. After cooling to room temperature, **1** (105.0 mg, 0.25 mmol), AgNO_3_ (7.0 mg, 0.04 mmol) and dry toluene (20.0 mL) were add into the reaction system, and then refluxed for 2 days. After cooling, solvents were removed under vacuum, and the black residue was subject to silica gel chromatography using n-hexane as eluent to afford **2** as a yellow powder (37.9 mg, 23%). [^2^](#_ENREF_2) A yellow single crystal can be obtained by recrystallization from CHCl_3_ and n-hexane as combined solvents. ^1^H NMR (400 MHz, CDCl_3_) δ(ppm): 8.05 (d, J = 1.8 Hz, 2H), 7.49 (dd, J = 8.9, 2.2 Hz, 6H), 7.18-7.04 (m, 8H), 4.05 (t, J = 7.2 Hz, 2H), 3.40-1.83 (w, 20H), 1.66 (p, J = 7.3 Hz, 2H), 1.25-1.19 (m, 2H), 0.87 (t, J = 7.3 Hz, 3H). ^13^C (600 MHz, CDCl_3_) δ(ppm): 159.3, 140.3, 132.9, 129.6, 124.0, 122.5, 116.0, 114.1, 114.0, 109.0, 89.2, 87.7, 55.3, 43.1, 31.1, 20.5, 13.9. ^11^B NMR (600 MHz, CDCl_3_) δ(ppm): -3.1 (4B), -11.2 (6B). IR (KBr): (ν cm^-1^) 2576 (B-H).

**3. Supporting figures**


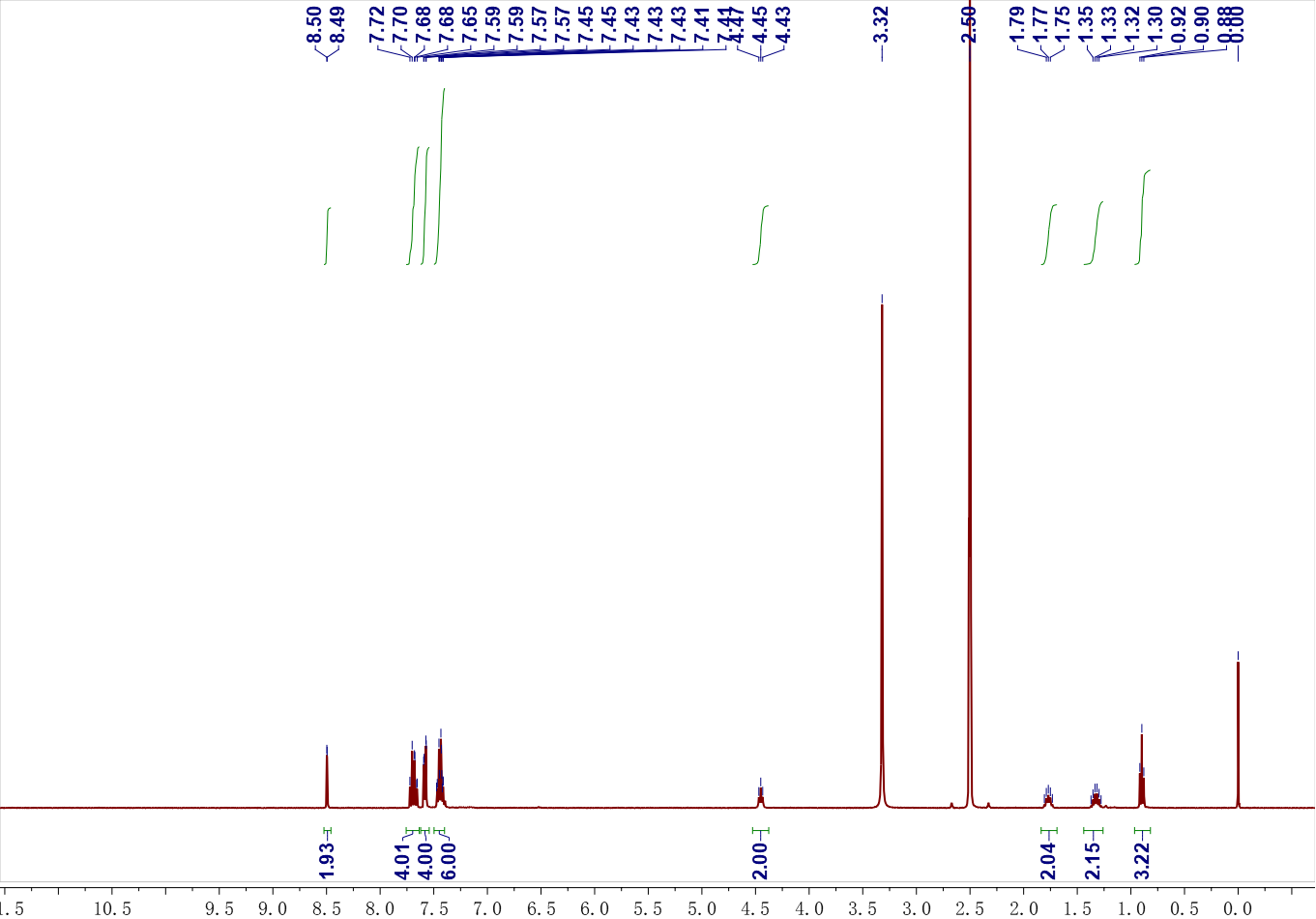


**Figure S1.** ^1^H NMR (400 MHz, *d*_6_-DMSO) of **1**.


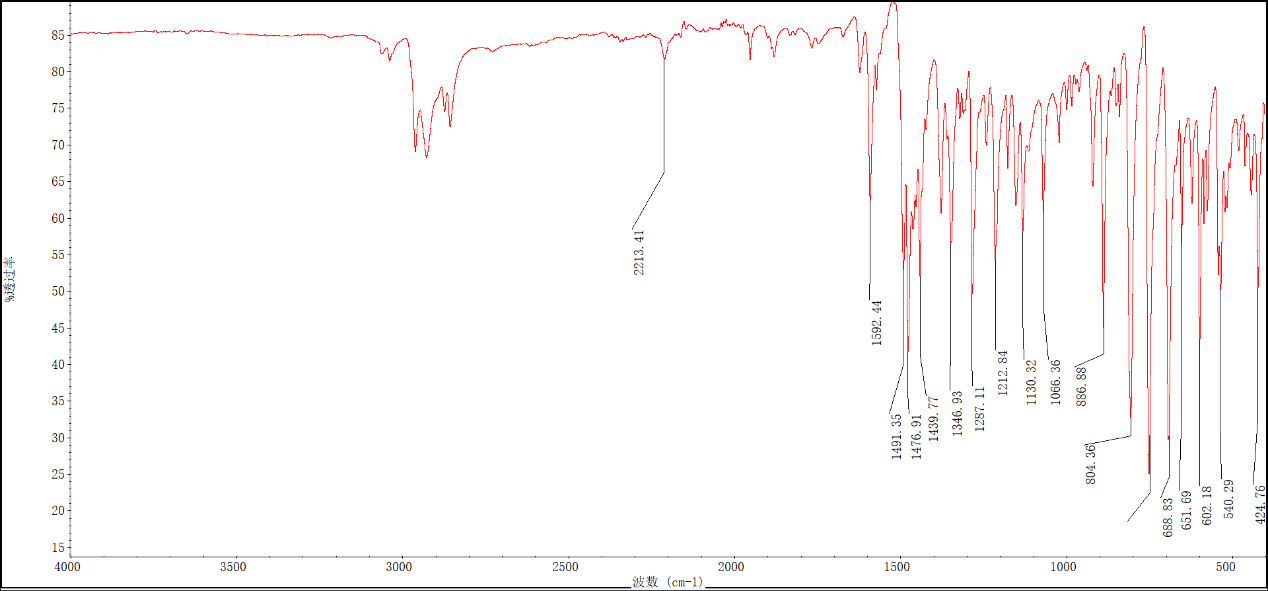


**Figure S2.** IR spectrum of **1**.


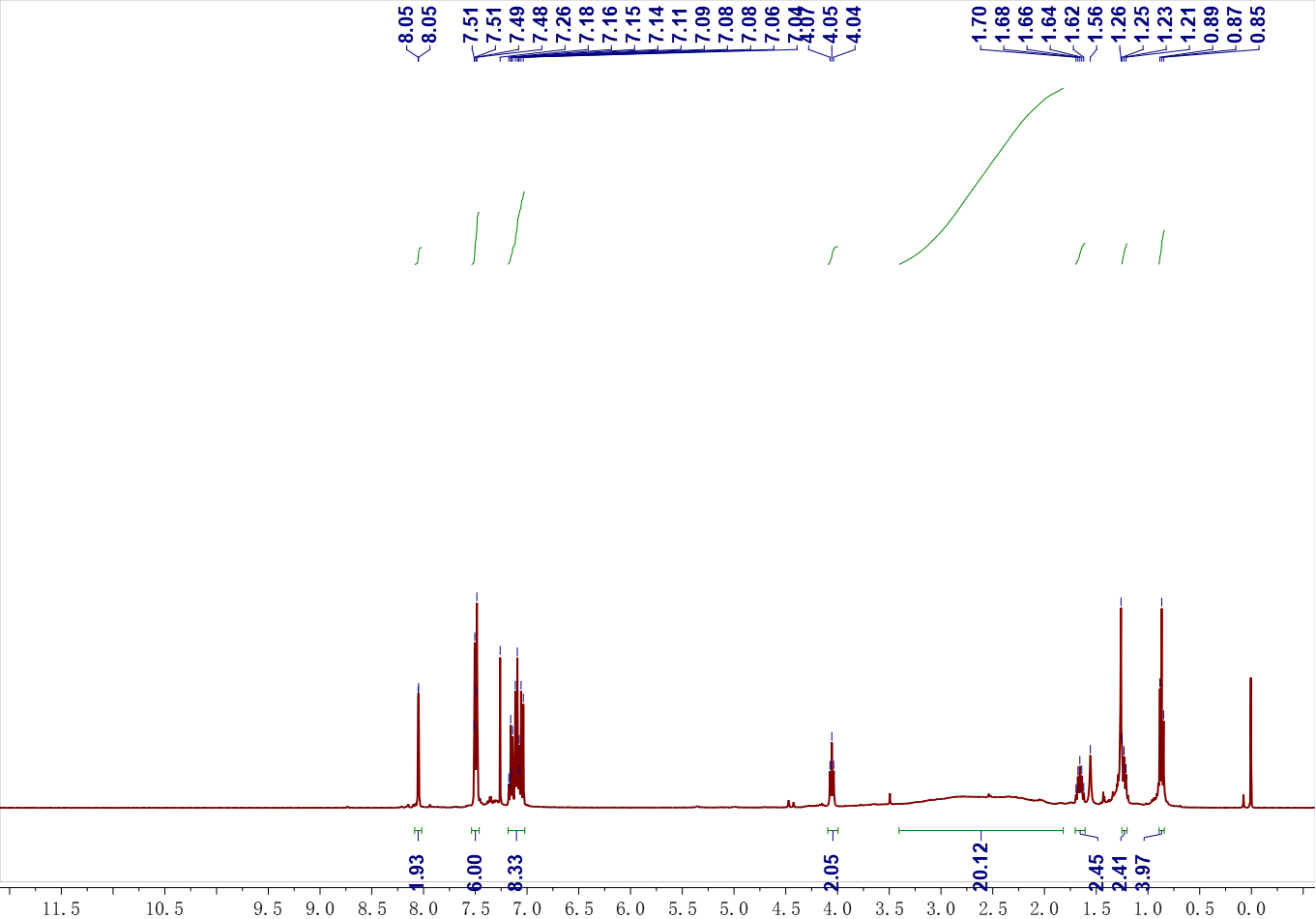


**Figure S3.** ^1^H NMR (400 MHz, CDCl_3_) of **2**.


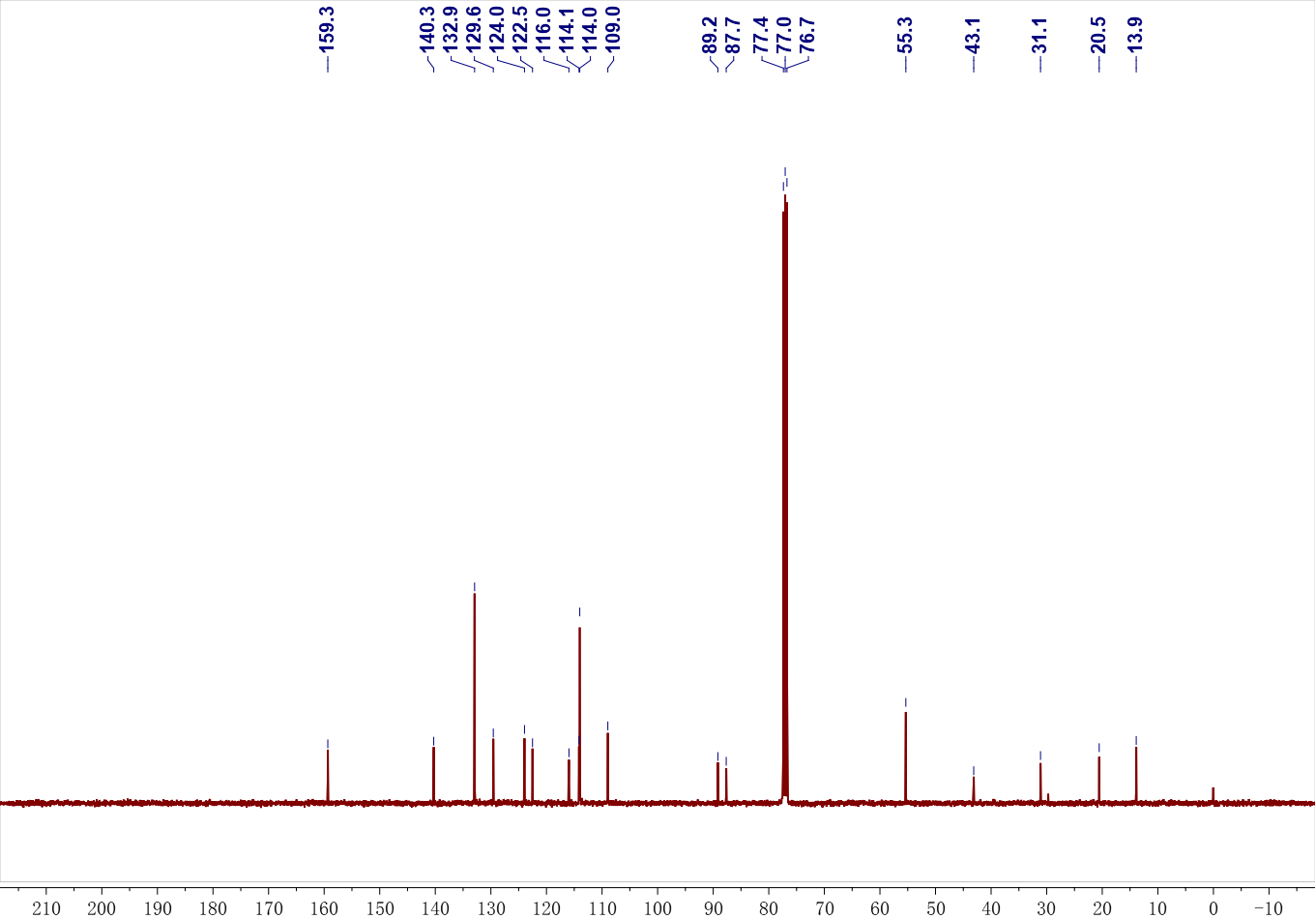


**Figure S4.** ^13^C NMR (600 MHz, CDCl_3_) of **2**.


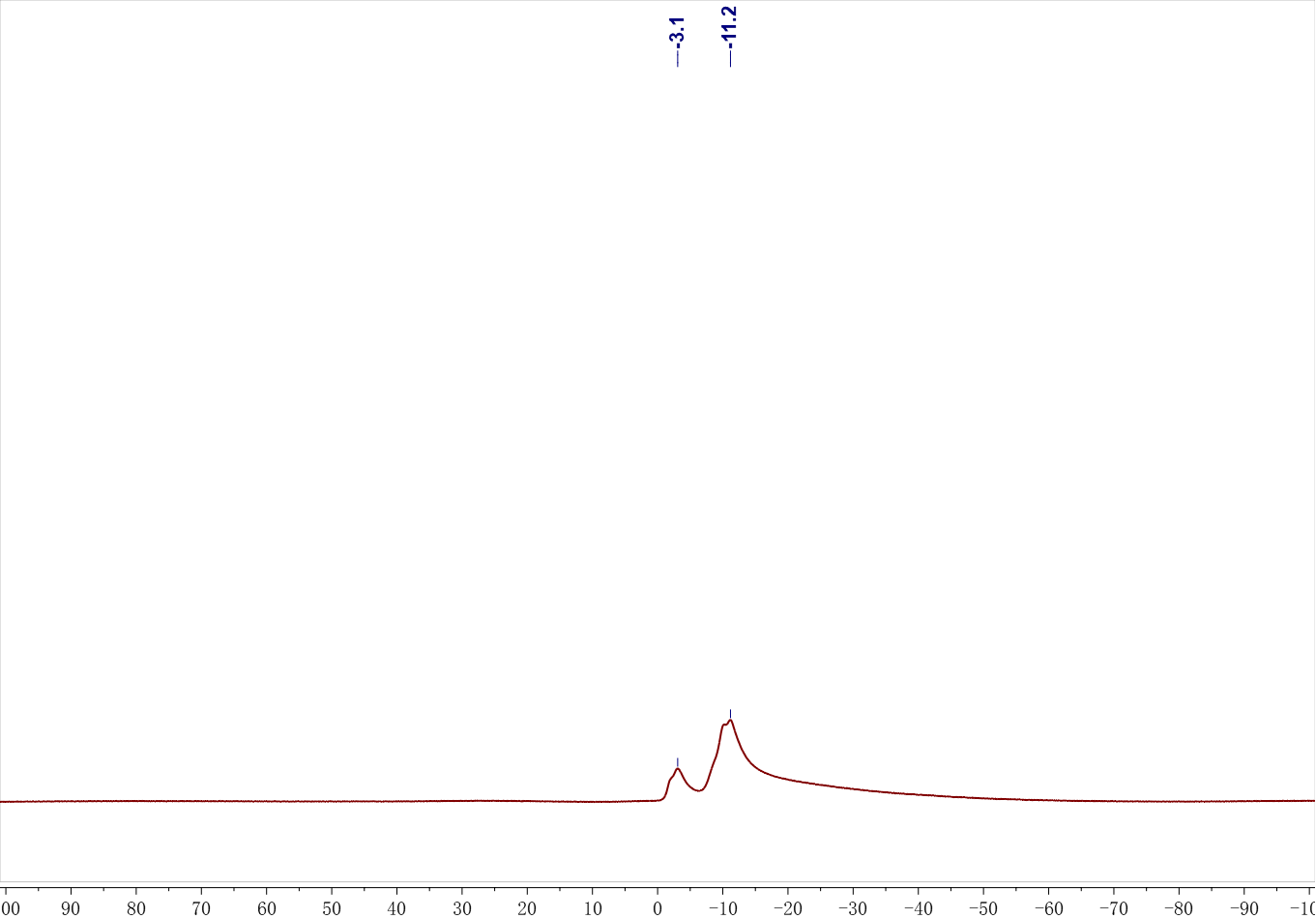


**Figure S5.** ^11^B NMR (600 MHz, CDCl_3_) of **2**.


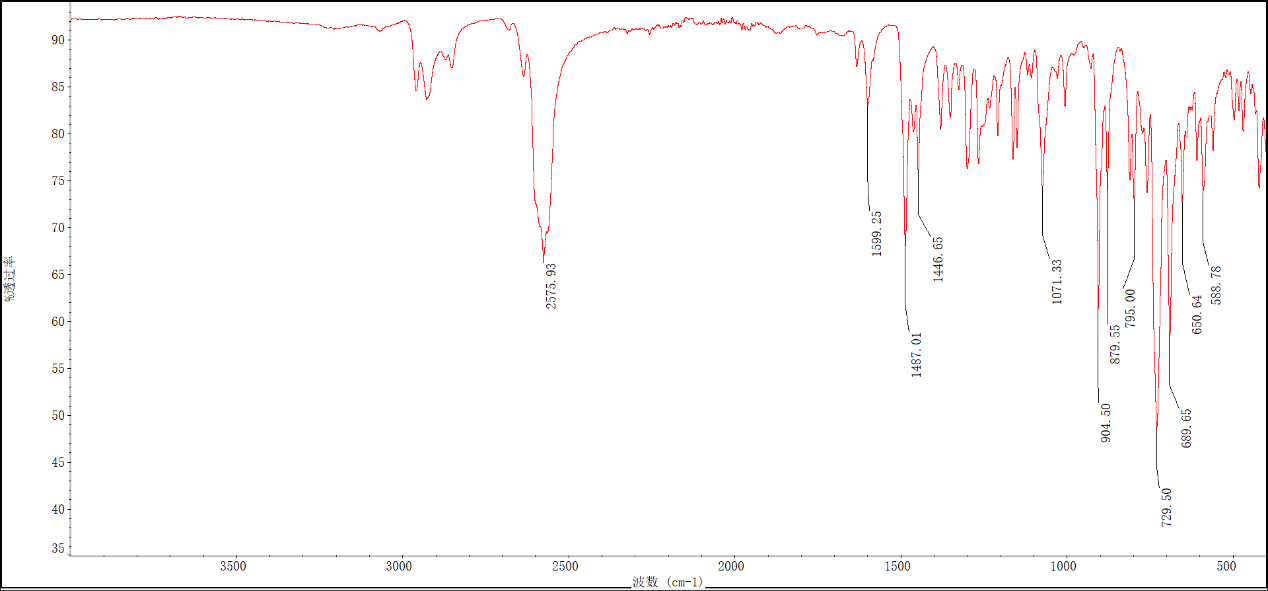


**Figure S6.** IR spectrum of **2**.

**4. X-ray structure determination**

X-ray diffraction data was collected on a Burker Smart CCD Apex DUO diffractometer with a graphite monochromated CuKα radiation (λ =1.54184 Å) using the ω-2θ scan mode. The data were corrected for Lorenz and polarization effects. The structure was solved by direct methods and refined on F^2^ by full-matrix least-squares methods using SHELXTL-2000. All calculations and molecular graphics were carried out on a computer using the SHELX-2000 program package and Diamond software.

**Table S1.** Crystal data and structure refinement detail for **2**.

| Compound | **2** |
| --- | --- |
| CCDC | 1900737 |
| Experimental formular | C_32_H_45_B_20_N, 1.5(CHCl_3_) |
| Formula weight | 838.44 |
| Temperature/K | 200(10) |
| Radiation/ Å | CuKα (λ = 1.54184) |
| Crystal system | triclinic |
| Space group | P-1 |
| a/Å | 10.4511(2) |
| b/Å | 13.2462(2) |
| c/Å | 16.0310(2) |
| α/° | 81.5640(10) |
| β/° | 83.3220(10) |
| γ/° | 83.5080(10) |
| Volume/Å^3^ | 2169.95(6) |
| Z | 2 |
| ρ_calc_g/cm^3^ | 1.283 |
| μ/mm^‑1^ | 2.961 |
| F(000) | 861.0 |
| 2θ range for data collection/° | 5.602 to 146.586 |
| Index ranges | -12 ≤ h ≤ 11, -16 ≤ k ≤ 15, -19 ≤ l ≤ 10 |
| Reflections collected | 17551 |
| Independent reflections | 8311 [R_int_ = 0.0517, R_sigma_ = 0.0698] |
| Data/restraints/parameters | 8311/12/551 |
| Goodness-of-fit on F^2^ | 1.082 |
| Final R indexes [I>=2σ (I)] | R_1_ = 0.1071, wR_2_ = 0.2542 |
| Final R indexes [all data] | R_1_ = 0.1280, wR_2_ = 0.2627 |
| Largest diff. peak/hole / e Å^-3^ | 1.65/-1.50 |


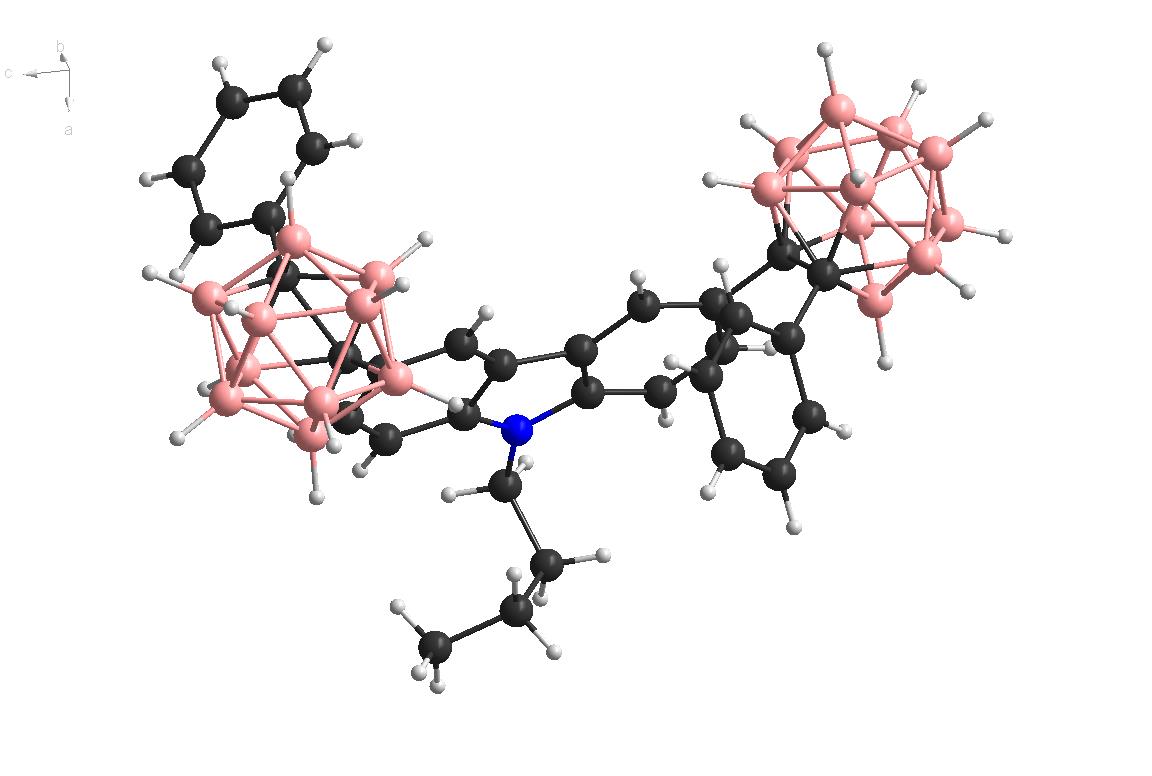


**Figure S7.** X-ray structure of **2**. Solvent residue CHCl_3_ were removed in this figure.

**5. FL spectra**

**Figure S8.** Fluorescence spectra of **1** at 1.0×10^-6^ mol/L in different solvents.

**Figure S9.** Fluorescence spectra of **2** at 1.0×10^-5^ mol/L in different solvents.

**Figure S10.** Variable temperature-fluorescence spectra of **2** at 1.0×10^-5^ mol/L in THF solution. Temperature range from 263 to 313 K.

**Figure S11**. a) Transient PL decay of **1** and b) residue of the bi-exponential decay fitting.

| **Table S2. Fitting statistics of bi-exponential decay for compound 1.** | | | | | | | | | | | | |
| --- | --- | --- | --- | --- | --- | --- | --- | --- | --- | --- | --- | --- |
|  | **y0** | | **A1** | | **t1** | | **A2** | | **t2** | | **Statistics** | |
|  | **Value** | **Standard Error** | **Value** | **Standard Error** | **Value** | **Standard Error** | **Value** | **Standard Error** | **Value** | **Standard Error** | **Reduced Chi-Sqr** | **Adj. R-Square** |
| **Decay** | **2.06624** | **0.47686** | **152671.02149** | **6303.81975** | **1.52001** | **0.02239** | **1377.46324** | **406.95503** | **4.18417** | **0.34415** | **165.61657** | **0.99819** |

**Figure S12**. a) Transient PL decay of **2** and b) residue of the mono-exponential decay fitting.

| **Table S3. Fitting statistics of mono-exponential decay for compound 2.** | | | | | | | | |
| --- | --- | --- | --- | --- | --- | --- | --- | --- |
|  | **y0** | | **A1** | | **t1** | | **Statistics** | |
|  | **Value** | **Standard Error** | **Value** | **Standard Error** | **Value** | **Standard Error** | **Reduced Chi-Sqr** | **Adj. R-Square** |
| **Decay4** | **16.54199** | **0.85765** | **15453.15438** | **45.78913** | **5.58967** | **0.01023** | **537.72509** | **0.99921** |

**References:**

1. Huangtianzhi Zhu, B. S., Kexian Chen, Peifa Wei, Danyu Xia, Julfikar Hassan Mondal, Feihe Huang, Cyclo[4]carbazole, an Iodide Anion Macrocyclic Receptor. *Org. Lett.* **2016,** *18* (19), 5054-5057.

2. Antonio Toppino, A. R. G., Mohamed E. El-Zaria, James Reeve, Fargol Mostofian, Jeff Kent, John F. Valliant, High yielding preparation of dicarba-closo-dodecaboranes using a silver(I) mediated dehydrogenative alkyne-insertion reaction. *Inorg. Chem.* **2013,** *52* (15), 8743-9.
